# Results from the translation and adaptation of the Iranian Short-Form McGill Pain Questionnaire (I-SF-MPQ): preliminary evidence of its reliability, construct validity and sensitivity in an Iranian pain population

**DOI:** 10.1186/1758-2555-3-27

**Published:** 2011-11-10

**Authors:** Farhad Adelmanesh, Ali Arvantaj, Hassan Rashki, Seyedmehdi Ketabchi, Ali Montazeri, Gholamreza Raissi

**Affiliations:** 1 Multidisciplinary Pain Clinic, Kasra Hospital, Tehran, 1514945311, Iran; 2 Department of Neurology, Baylor College of Medicine, 1 Baylor Plaza, Houston, 77030, USA; 3 Department of Community Medicine, Shahid Beheshti University of Medical Sciences, Daneshjoo Street, Tehran, 1966833831, Iran; 4 Mental Health Research Group, Mother and Child Health Research Centre, Iranian Institute for Health Sciences Research, ACECR, Tehran, 13185, Iran; 5 Department of Physical Medicine and Rehabilitation, Tehran University of Medical Sciences, Firoozgar Hospital, Behafarin Street, Tehran, 1593748771, Iran

**Keywords:** Short Form McGill Pain Questionnaire, Iranian version, Pain assessment, Reliability, Construct validity

## Abstract

**Background:**

The Short Form McGill Pain Questionnaire (SF-MPQ) is one of the most widely used instruments to assess pain. The aim of this study was to translate and culturally adapt the questionnaire for Farsi (the official language of Iran) speakers in order to test its reliability and sensitivity.

**Methods:**

We followed Guillemin's guidelines for cross-cultural adaption of health-related measures, which include forward-backward translations, expert committee meetings, and face validity testing in a pilot group. Subsequently, the questionnaire was administered to a sample of 100 diverse chronic pain patients attending a tertiary pain and rehabilitation clinic. In order to evaluate test-retest reliability, patients completed the questionnaire in the morning and early evening of their first visit. Finally, patients were asked to complete the questionnaire for the third time after completing a standardized treatment protocol three weeks later. Intraclass correlation coefficient (ICC) was used to evaluate reliability. We used principle component analysis to assess construct validity.

**Results:**

Ninety-two subjects completed the questionnaire both in the morning and in the evening of the first visit (test-retest reliability), and after three weeks (sensitivity to change). Eight patients who did not finish treatment protocol were excluded from the study. Internal consistency was found by Cronbach's alpha to be 0.951, 0.832 and 0.840 for sensory, affective and total scores respectively. ICC resulted in 0.906 for sensory, 0.712 for affective and 0.912 for total pain score. Item to subscale score correlations supported the convergent validity of each item to its hypothesized subscale. Correlations were observed to range from r^2 ^= 0.202 to r^2 ^= 0.739. Sensitivity or responsiveness was evaluated by pair t-test, which exhibited a significant difference between pre- and post-treatment scores (p < 0.001).

**Conclusion:**

The results of this study indicate that the Iranian version of the SF-MPQ is a reliable questionnaire and responsive to changes in the subscale and total pain scores in Persian chronic pain patients over time.

## Introduction

Pain is the most common reason for patients seeking medical attention; but due to the inherent nature of pain, being a subjective personal experience spanning both physical and emotional consequences, assessment and treatment of pain disorders is challenging. Although scales like numerical rating, verbal rating and visual analogue scale (VAS) have been frequently and successfully used in pain sensation intensity recording, they lack the ability to assess the qualitative aspects of this personal experience [1-3]. Melzack and Torgerson began specifying qualitative descriptors of pain in 1971. Their study led to the development of the McGill Pain Questionnaire (MPQ) in 1975 [1]. The MPQ has been one of the most widely used tools for more than 30 years, and is designed to assess sensory, affective and evaluative dimensions of pain [4]. It has been translated and used in many different languages such as: Dutch, French, and German [5-8]. It has also been used in numerous studies with diverse patient samples [9,10]. Despite the fact that the MPQ usually takes less than 20 minutes to administer, this time is not always logistically possible in some groups like cancer patients because they are unable to concentrate for a prolonged period of time [11]. The other problem with the MPQ is that it includes excessive detail, parts of which may be unnecessary and time-consuming in certain therapeutic trials.

Taking these aspects into consideration, Melzack developed the short form of the MPQ (SF-MPQ) in 1987. The SF-MPQ was developed to collect data from patients in a short time, when more information rather than intensity measures such as VAS and Present Pain Intensity scale were needed. It has similar MPQ properties but takes less time [12], so it can be used in a routine clinical environment [13]. The SF-MPQ has been used in several studies of chronic pain, like low back pain [14], fibromyalgia syndrome [15], osteoarthritic pain [16,17], neuropathic pain [18] and acute pain with diverse etiology [7,19-21]. The SF-MPQ has been translated into many languages, including Czech [22], Swedish [23], Greek [4], Korean [24], Turkish [25] and Norwegian [26].

Farsi is the official language spoken in Iran, Afghanistan and Tajikistan, and is widely used in Uzbekistan. Moreover, it is spoken to some extent in Iraq, Bahrain, Oman and Kazakhstan as well as large communities in the US (413845) and Canada (121510), according to the US and Canada Census of 2006. As a result of new waves of immigrations to the US and Canada since then, it may be reasonable to assume that these numbers have increased. Finally, in diverse ethnic groups in different parts of Iran whose native languages include Turkish, Kurdish, Baluchi and Arabic, Farsi is the *lingua franca *of most of these groups [27-30].

The aim of this study was to translate and culturally adapt the short form of the McGill Pain Questionnaire into Farsi, the official language of Iran, in order to make an easily understood, faithful, reliable, valid and sensitive translation of SF-MPQ to be used as a tool to assess pain in the Iranian population. The new measure, the Iranian Short-Form McGill Pain Questionnaire (I-SF-MPQ), was tested for its reliability and responsiveness in chronic pain patients.

## Methods

Guillemin's guidelines for cross-cultural adaptation of health related questionnaires was used [31,32]. Two official translators translated the SF-MPQ to Farsi, then it was discussed in a committee of translators and physicians expert in pain management, including physical medicine and rehabilitation, anesthesiology, neurology and oncology. Moreover, we consulted with other physicians managing patient's pain in gynecology, orthopedics and neurosurgery; they were ethnically heterogeneous self-identifying as Persians, Turks, Kurds, Arabs and Baluchs. After reaching consensus on translated words, two other English-speaking translators, who were totally blind to the original questionnaire, translated the Iranian version back to English. The divergence between the translations was discussed and resolved in the expert committee of physicians and consultants. As a preliminary test, the pre-final version of the questionnaire was administered to 30 chronic pain patients. If we observed, during this preliminary testing, that some words were difficult for patients to comprehend, we provided short descriptions to help better describe the pain qualities.

### Instrument

The SF-MPQ consists of three parts. The main component consists of 15 descriptive adjectives, 11 sensory and four affective, which are rated by the patients according to their severity on a four point scale (0 = none, 1 = mild, 2 = moderate, 3 = severe), yielding three scores. The sensory and affective scores are calculated by adding sensory and affective item values separately, and the total score is the sum of the two above-mentioned scores.

The second part is the VAS, which is a 10-centimeter horizontal line with clearly defined boundaries with descriptive anchors ranging from "no pain" to the "worst possible pain". The intensity of pain was calculated from point zero to the point where the patient had marked in centimeters, and represented the intensity of pain at the time of completing the questionnaire.

**Figure 1:**
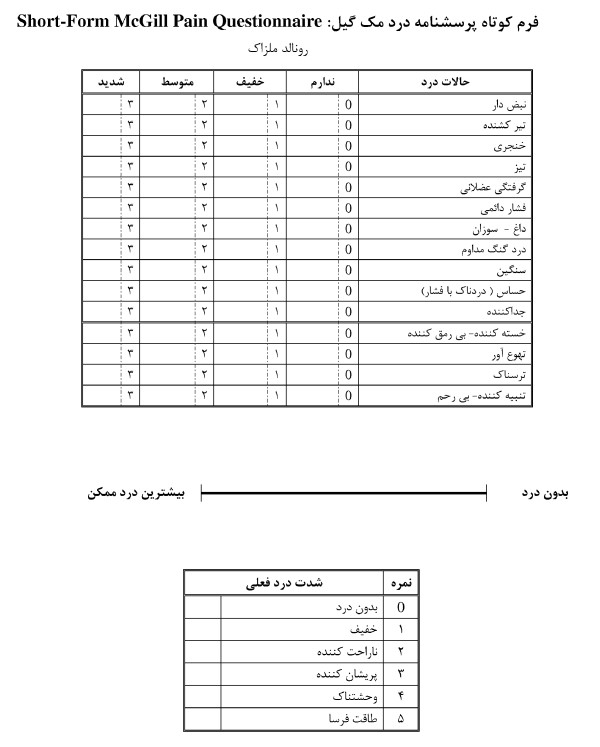


The third part of the SF-MPQ is present pain intensity (PPI), which is a six-point verbal rating scale. In this scale, patients were asked to choose between six words, from none (0) to the worst excruciating (5); choosing the word that best describes the overall intensity of their pain at the time of completing the questionnaire (Figure 1).

### Patients

We used a convenience sampling method for case selection from a diverse group of chronic pain patients who were referred to our tertiary pain and rehabilitation clinic over three months. The inclusion criteria were: having chronic pain (more than three months), age over 18, ability to speak and understand Farsi, and a willingness to sign the consent form and participate in the study. The exclusion criteria were: intellectual disability, psychosis, and dementia.

Among the patients referred to our Pain Clinic supported by the department of Physical Medicine and Rehabilitation, the first 100 patients who fulfilled above criteria were asked to complete the questionnaire three times; the first time (T1) was on the morning of their first visit (between 11 am & 1 pm); the second (T2) was on the evening of the same day (between 5:00 and & 7:00 pm); the third time (T3) was three weeks later. The subjects did not receive any treatment or intervention between T1 and T2. Eight patients who had not completed the questionnaire all three times were excluded. All subjects underwent a three-week supervised multidisciplinary rehabilitation program which included a combination of education, relative rest, relaxation, stretching, exercise, case specific physical modalities and pain medications based on their problem. The questionnaire was delivered to the patients all three times by the same assessor. An assistant read the questionnaire for three illiterate patients to fill out the items. An informed consent was obtained from all patients.

### Statistical analysis

All statistical analysis was completed using the SPSS 17 (Chicago, IL). The demographic data of the patients were described by the use of mean and standard deviations. Alpha was set at 0.05 to indicate a statistical significance. Cronbach's alpha was used to assess internal consistency of the I-SF-MPQ. Test-retest reliability was assessed by means of the ICC. The principle component analysis (PCA) was done to assess construct validity. Sensitivity or responsiveness to change was assessed by pair t-test analysis.

## Results

### Demographics

From 100 patients, 92 of the patients finished the standardized treatment protocol, and completed the questionnaire after three weeks. Patients were 66 (71.7%) women and 26 (28.2%) men, with a mean age of 45.2 ± 15.6 years. 4.3% had elementary (6 years) education, 44.5% completed high school and 47.8% had a university degree. Housewives constituted 34.8% of the participants. The patients comprised a diverse group of musculoskeletal pain (knee and neck osteoarthritis, lower back pain, fibromyalgia syndrome) carpal tunnel syndrome and other types of radiculopathic pain. The demographic data are shown in Table 1.

**Table 1:** Demographic characteristic of the participants

		Frequency
Gender		
	Male	26 (28.3%)
	Female	66 (71.7%)
Marital status		
	Single	15 (16%)
	Married	71 (77%)
	Divorced/Widowed	6 (7%)
Education		
	Illiterate	3 (3.2%)
	Elementary	4 (4.3%)
	First high school	7 (7.6%)
	Second high school	34 (36.9%)
	University degree	44 (47.8%)
Employment status		
	Housewife	32 (34.7%)
	Student	1 (1.0%)
	Employed	26 (28%)
	Unemployed	9 (9.7%)
	Other	8 (8.6%)
	Unknown	16 (17.3%)

### Item selection and translation reconciliation

The following are some words that proved difficult during translation:

1. "Throbbing": on translation of this word, we were in doubt to choose between "pulsating" []vs. "beating" []. Due to a physician's expertise and asking 10 patients with throbbing pain which word they prefer to describe their pain, "pulsating" was chosen.

3. "Stabbing": In the Farsi language, there are two synonyms for this word: "got hit by a knife" or "got hit by a dagger"; both experts committee and patients preferred one of these meanings: "got hit by a dagger" [].

4. "Sharp": all agreed on the word [], a pain like the tip of a knife with clear boundaries.

6. "Gnawing": was the most controversial word of all. The back translation of synonyms would be: "continuous pressure", "chewing", "erosive", and "pressing". The meaning, "continuous pressure", []was chosen.

8. "Aching": the back translation synonym means a "continuous dull pain", [].

10. "Tender": in order to differentiate it from Allodynia, we decided to add a short expression in parenthesis, "sensitive (painful by pressure)" [].

13. "Sickening": the back translation synonym means, "pain that causes nausea", [].

### Reliability

The internal consistency of the test using Cronbach's alpha was found to be 0.951 for sensory, 0.832 for affective, and 0.840 for total scores. For test-retest reliability, the ICCs were found to be 0.906 for sensory, 0.712 for affective, and 0.912 for total scores (Table 2).

**Table 2:** Internal Consistency and Test-retest Reliability of Iranian version of Short-Form Mc Gill Pain Questionnaire

	Number of items	T1 Mean ± SD	T2 Mean ± SD	Chronbach's alpha Coefficient (T1, T2)	ICC
Sensory	11	8.03 ± 5.99	7.95 ± 5.45	0.951, 0.940	0.906
Affective	4	2.64 ± 2.78	2.51 ± 2.89	0.832, 0.809	0.712
Total	15	10.60 ± 7.88	10.67 ± 7.45	0.840, 0.837	0.912
VAS	2	4.43 ± 2.57	4.39 ± 2.61	0.870*	0.870
PPI	2	2 ± 1.05	1.99 ± 1.23	0.820*	0.820

T1 First Questionnaire completion attempt, T2 second questionnaire completion (evening of the same day), VAS visual analogue scale, PPI present pain intensity

* T1 vs. T2

### Validity

The PCA and oblimin rotation were used to evaluate the construct validity. Correlation between scores of each sensory and affective word was compared with the total number of sensory and affective words. It was shown that sensory words had more correlation with total sensory scores compared with total affective scores. However, this difference is significant only for the word "throbbing", which shows this word can clearly differentiate sensory words from affective words. This kind of consideration was used for affective words as well; overall, their correlation with total affective scores was more than their correlation with total sensory scores (Table 3).

**Table 3:** Principle component analysis of the Iranian version of the Short-Form McGill Pain Questionnaire in a diverse chronic pain patient sample

	Factor 1 (Sensory)	Factor 2 (Affective)
Items	r	r
1. Throbbing	0.47	0.11
2. Shooting	0.68	0.45
3. Stabbing	0.59	0.26
4. Sharp	0.64	0.31
5. Cramping	0.45	0.29
6. Gnawing	0.69	0.54
7. Hot-Burning	0.55	0.39
8. Aching	0.48	0.44
9. Heavy	0.64	0.43
10. Tender	0.58	0.40
11. Splitting	0.52	0.25
12. Tiring-Exhausting	0.49	0.75*
13. Sickening	0.52	0.86*
14. Fearful	0.41	0.66*
15. Punishing-Cruel	0.46	0.82*

r = Correlation Coefficient

*More prominent in Second Factor

### Sensitivity

Ninety-two of the patients who had filled the questionnaire first day finished standardized treatment protocol in three weeks. A paired analysis t-test was used to evaluate the I-SF-MPQ as a sensitive questionnaire to compare the pre- and post-treatment changes in pain scores. As the results show in Table 4, there was a statistically significant difference (p < 0.001) between the pre- and post-treatment scores. There was also a significant decrease in the mean pain scores in all the subclasses. These results show that the I-SF-MPQ is a sensitive tool to evaluate treatment efficacy (Table 4).

**Table 4:** Sensitivity measurement; Pair T test analysis of pre and post-treatment scores

	Pretreatment (n = 92)	Post treatment (n = 92)	P value
Sensory	*8.03 ± 5.99	7.45 ± 5.45	< 0.0001
Affective	2.64 ± 2.78	2.31 ± 3	< 0.0001
Total	10.60 ± 7.88	9.78 ± 7.45	< 0.0001
VAS(cm)	4.43 ± 2.57	4.29 ± 2.60	< 0.0001
PPI	2 ± 1.05	1.90 ± 1.23	< 0.0001

*Mean ± SD

## Discussion

In this era of globalization and exchanging data, measurements should be comparable in different countries with language and cultural diversity. Translation of existing health care scales appears to be a logical, efficient, and popular approach to produce comparable tools. Translation of existing western measures of health status is challenging. There are complex variations in perception of health versus disease and describing symptoms in different cultures. As a result, translation and adaptation is a rigorous and step-wise process to ensure that questionnaires are comparable. The most important requirements of a translated measure are that it be useful, valid, consistent, reliable, and sensitive [33].

In this study, three different tests were used to evaluate the I-SF-MPQ. The Cronbach's alpha coefficient for internal consistency (IC) was 0.951, 0.832 and 0.840 for sensory, affective and total scores respectively, demonstrating higher than [2] or similar IC estimate to other translated versions [23-26]. The test-retest reliability was excellent; for sensory 0.906, affective 0.712 and total 0.912, which was comparable with other versions [2,23-25]. Patients in this study had different pain etiologies, such as rheumatic and musculoskeletal pain. In previous studies [4,25], total ICC in musculoskeletal pain participants was reported about 0.75, and in rheumatic patients about 0.96; therefore, it seems that the ICC in this study (0.912) is acceptable and concordant with previous studies.

To evaluate the construct validity of the questionnaire, we used the PCA (Table 3). Each sensory word score was highly correlated with Factor 1 (Sensory), while each affective word score showed higher correlation with Factor 2 (Affective), supporting the construct validity of the Iranian version of the SF-MPQ. The PCA showed a cross loading of these words: "gnawing", "aching", "tender", and "sickening", in both the sensory and affective components. The first three words have a dominant relation in the sensory component, and "sickening" shows a dominant relation to the affective component. Although it is not uncommon in the process of questionnaire translation and validation, the Farsi words that were chosen for translation of these items have mixed emotional and body sense structure and might lead to cross loading. If different words had been chosen, this problem might have been resolved. Different factorial structures have been reported in some previous studies. For example, the Swedish version of the SF-MPQ was known as a three-factorial model [23]. Beattie reported a model of the SF-MPQ in chronic lower back pain patients which was different from Melzack's model. This model showed a sensory and sensory-affective component that was referred to as the modified SF-MPQ [34].

Inaccurate translation of items may change the factorial structure of a questionnaire, but this factorial difference has been reported as a result of cultural differences. Zinke and colleagues examined the cross-cultural validity of the English version of the SF-MPQ [35]. They found three factorial components during the preliminary PCA of the Hispanic patients' data and four factorial components from the non-Hispanic patients. They finally selected two factorial structures for each group, but the minimum level of loading was more than 0.4 for the Hispanic and more than 0.5 for the non-Hispanic participants. In addition, the final factorial components were different between groups. The first factor in the Hispanic group consisted of "sharp", "stabbing" and "shooting" pain descriptors, and the remnant descriptors were located as the second factor component. In the non-Hispanic group, "tender", "burning-hot", and "throbbing" in addition to three mentioned items formed first component. The results of this study are different from Melzac's study on the English version [12]. It seems that the factorial analysis can lead to different components based on the characteristics of participants' pain and ethnicity.

Difference in the pre and post-treatment mean total score in our patients was clinically non-significant, but the decrease was statistically significant in all items. This sensitivity indicates that an instrument can be used to evaluate small improvements in chronic pain diseases, such as osteoarthritis, in which usually pain changes are incremental. The ability to discriminate improved and non-improved patients with musculoskeletal pain in the Norwegian version [36] was also low (61%). The VAS is a good scale with no need to translate and can be used as a reliable score of pain intensity. In our study, the post-treatment reduction in the VAS was correlated with an overall reduction of the I-SF-MPQ score.

One of our limitations is the lack of a parallel scale as a gold standard to validate treatment results with the I-SF-MPQ scores. Validated quality of life questionnaires or objective activity measurement scales are suitable for this purpose. The second limitation was the selection of chronic pain patients. The response to treatment is usually gradual in these patients; therefore, they are suitable for a test-retest evaluation instead of an evaluation of responsiveness to change. We should have planned at least a two-month treatment regimen to be able to use the SF-36 as a quality-of-life questionnaire.

Our study revealed a high test-retest reliability and construct validity. Most of the previous studies have used a restricted group of patients, such as osteoarthritis patients [17], lower back pain [34], or patients with rheumatoid arthritis [25]. We chose a diverse group of chronic pain patients to test the reliability and validity of questionnaire in a wide range of patients. Participation of different ethnicities in our translation team improved our ability to select comprehensible words for patients from different regions of the country.

Further assessment of the I-SF-MPQ reliability, validity and sensitivity should be done in patients with acute pain. The discriminative capability of the I-SF-MPQ should be examined in different pain conditions.

## Conclusion

In conclusion this study supports the use of the Iranian version of the SF-MPQ as a reliable, valid and sensitive multidimensional measure to assess musculoskeletal pain in Farsi speaking patients. It can be used by Iranian clinicians and researchers working in the field of pain to communicate internationally.

## Competing interests

The authors declare that they have no competing interests.

## Authors' contributions

FA was the main investigator and analyzed the data, wrote the first draft, and revised the final manuscript. AA and HR contributed to the study design and the analysis. SMK contributed to the acquisition of data. GR helped as a consultant in all parts of the study, especially proposal writing, data analysis, and revising manuscript. All authors read and approved the final manuscript.
